# Dr. P. T. Y.

**Published:** 1873-07

**Authors:** 


*Dr. P. T. Y.*

In reply we will state, that while we "are for peace," and "as much as within us lies" mean to "preserve it," still we hold our journal open for the discussion of all questions of science and ethics having in view the interests of medicine and the welfare of the profession. Scientific discussion based upon differences of opinion can not possibly be injurious, but, on the contrary, highly beneficial, if the proper regard both in language and matter be observed and maintained.

